# ‘A Burden on the County’: Madness, Institutions of Confinement and the Irish Patient in Victorian Lancashire

**DOI:** 10.1093/shm/hku082

**Published:** 2015-01-07

**Authors:** Catherine Cox, Hilary Marland

**Keywords:** asylums, workhouses, Irish, patients, chronic, overcrowding, cost

## Abstract

This article explores the responses of the Poor Law authorities, asylum superintendents and Lunacy Commissioners to the huge influx of Irish patients into the Lancashire public asylum system, a system facing intense pressure in terms of numbers and costs, in the latter half of the nineteenth century. In particular, it examines the ways in which patients were passed, bartered and exchanged between two sets of institution—workhouses and asylums. In the mid-nineteenth century removal to asylums was advocated for all cases of mental disorder by asylum medical superintendents and the Lunacy Commissioners; by its end, asylum doctors were resisting the attempts of Poor Law officials to ‘dump’ increasing numbers of chronic cases into their wards. The article situates the Irish patient at the centre of tussles between those with a stake in lunacy provision as a group recognised as numerous, disruptive and isolated.

In Lancashire, as in most manufacturing counties and districts, the material we get to work upon is bad; that is to say the graver forms of insanity prevail, and we receive a large proportion of broken-down cases. We can do some good even with the worst material, but as there can be no question of selecting patients here the results, as far as cure is concerned, do not appear as brilliant as they might do if we had a larger proportion of curable patients to begin with.^1^

In the final three decades of the nineteenth century, statistics produced by the Lunacy Commissioners demonstrated an alarming increase in the number of pauper lunatics confined in lunatic asylums and workhouses in England and Wales.^2^ An impressive body of historical research has sought explanations for this increase, and the associated ‘rise of asylumdom’ in the second half of the nineteenth century.^3^ This work has illuminated the pivotal role of the English Poor Law in the administration of pauper lunacy and also revealed its significance as a ‘feeder’ of patients into asylums. Simultaneously, our understanding of the function of nineteenth-century workhouses has been recast somewhat as they emerge from this literature as important sites for the management of the mentally ill. Peter Bartlett has identified collaborations between Poor Law and asylum officials in the administration of lunacy legislation and in the supervision of population intake and institutional exchanges. While for Bartlett there was an alignment of interests, Joseph Melling, Richard Adair and Bill Forsythe's study of Devon's lunacy provision uncovered conflict and tension between Poor Law and lunacy administrators and asylum superintendents. Their work also emphasised the diversity of regional experiences and the significance of local contexts.^4^ Chris Philo, meanwhile, has pointed out the ambiguities resulting from different interpretations of Poor Law legislation, in terms of the process of decanting pauper lunatics to asylums. The General Workhouse Rules issued by the Poor Law Commissioners in 1842 stemmed from the 1834 Poor Law Amendment Act (Section 45), and indicated that persons of unsound mind, but not dangerous, could legally be kept in the workhouse, leaving a potential ambiguity in interpretation that was to be exploited by Poor Law guardians. The Rules also noted that it was ‘inappropriate’ to detain curable lunatics in workhouse accommodation and suggested that harmless incurables should be removed from the workhouse if specialist accommodation was available.^5^ At times, workhouse and asylum managers claimed authority to cater and care for the mentally ill, but as pressures mounted in terms of numbers, both groups expressed a reluctance to open their doors to the flood of pauper lunatics overwhelming their institutions.

## The ‘Irish Problem’

Bartlett and Adair, Melling and Forsythe have described the movements of patients from workhouse to asylum and back again, focusing on the actions of the medical officers and officials who administered the two systems of care and providing examples of individual patient careers.^6^ Here we focus on one patient group, Irish migrants, who contributed significantly to workhouse and asylum populations wherever they travelled in the nineteenth century and who were described by the local authorities—magistrates, Poor Law administrators and asylum managers—as being particularly adept in accessing welfare institutions. The Poor Law in England, whilst hardly generous, was easily accessed by Irish migrants, who utilised both workhouses and asylums as sites of economic survival. This was particularly so in Lancashire, and even more so in the port of Liverpool, which absorbed enormous numbers of Irish migrants during and in the decades following the Great Famine (1846–55). According to census returns, the Irish-born population of Lancashire nearly doubled between 1841 and 1851 reaching over 191,000; 10 per cent of the county's inhabitants.^7^ In Liverpool across the same decade, the Irish-born population rose from 49,639, 17.3 per cent of the total population, to 83,813 or 22.3 per cent.^8^ This movement of people was largely unregulated and those travelling to Liverpool during the Famine years typically arrived in very poor physical condition. The majority of Famine Irish entered low paid employment or became reliant on poor relief and tended to congregate in the worst districts of Liverpool, notable for their disgusting housing conditions and terrible overcrowding. By the late nineteenth century, this profile of the impoverished Irish was less accurate, and recent migrants and more established Irish settlers included middle-class, artisan and professional elements.^9^ For our purposes, however, we are mainly concerned with those on the margins of poverty who entered the public asylums and workhouses of Lancashire in the second half of the nineteenth century.

The burden of the Irish on the Lancashire Poor Law system prompted frequent commentary in Lancashire newspapers from the 1840s, reflecting and in many instances magnifying public anxieties. The initial sympathy expressed for the Famine migrants quickly transformed into fear, panic and outrage as the scale and potential cost of the suffering and starvation became apparent. As residents of a major port city in close proximity to Ireland, the population of Liverpool was especially apprehensive and local ratepayers rapidly came to resent the huge influx of Irish, as large numbers became dependent on the Poor Law during the humanitarian crisis of the Great Famine.^10^ Ratepayers believed that they were shouldering a disproportionate share of the tax burden in supporting Irish paupers, who benefited from their entitlement to relief under the English Poor Law. It is impossible to establish with accuracy the amounts spent on the relief of pauper Irish migrants, as much of this involved emergency aid when they arrived in Liverpool—food rations and accommodation in vagrant sheds. It was estimated that in 1847–48 49 per cent of all Liverpool Select Vestry's outdoor relief, some £20,750, was spent on Irish migrants.^11^ The clerk to Liverpool's Select Vestry, however, came up with a much higher figure, claiming that upwards of £70,000 was spent in these years on the relief of Irish migrants.^12^ Such relief involved various forms of expenditure: in-door and out-door relief, the cost of ‘passing’ back migrants to Ireland, the maintenance of fever sheds and the provision of vagrant sheds, which endured well beyond the Famine, and, soon to become one of the most expensive items, the accommodation of those suffering from mental disorders.

The cost of supporting Irish paupers in receipt of outdoor and indoor relief continued to drain the resources of the Lancashire Poor Law Unions long after the Famine, although this varied between unions. In February 1852, the editors of the *Preston Guardian*, which was particularly vociferous in its depiction of the Irish problem, described how ‘The miserable and demoralised crowds sent from Ireland into Liverpool, partly help to increase the Irish colonies already too extensively rooted here. … The Irish tramping through that district, or casually employed in “potato getting”, give it a character from which it would be otherwise free’.^13^ During economic crises, such as the Lancashire Cotton Famine (1861–65), the usefulness of Irish labour was questioned as they contributed to the pool of surplus labourers, and the Irish more generally were accused of depressing wages and strike-breaking.^14^ Yet, in better times hostility was tempered by the realisation that the Irish were an important source of labour for local industries and willing to take on work that no one else wanted.^15^

The Irish were depicted as presenting other threats to the well-being of the county through their association with public disturbances and criminality. They were held responsible for the outbreak of a variety of contagious diseases, including the typhus and cholera epidemics that struck in each decade from the 1840s through to the 1870s.^16^ In 1842, Dr W. H. Duncan, soon to be appointed Liverpool's first Medical Officer of Health, had noted the Irish disinclination for removal to hospital when struck down by fever, which led to the further spread of the disease, and remarked on their habits of keeping pigs in cellars and even garrets, ‘as well as the objectionable custom of retaining the bodies of the dead … in the sleeping rooms of the living’.^17^ Thus, disease was related to the behaviour of the Irish as much as to the poor environments they inhabited. Indeed, it was asserted that the Irish were naturally drawn to low living and overcrowded courts and streets where they congregated with their own kind. During a public meeting held in Liverpool in May 1847 a number of speakers urged provision for the relief of the Irish in Ireland to limit the impact on Lancashire. Liverpool magistrate Edward Rushton claimed that the Irish had pushed up mortality figures, their wretched living conditions resulting in the deaths from fever of ‘Catholic priests’ and ‘brave overseers’ alike. Another contributor to the debate expressed concern that the Irish would infect the English with their cultural habits, and ‘bring down the poor of England to their own level in poverty and, what was worse, in habits the very reverse of the spirit and cleanly habits of the English poor’.^18^

Although an impressive volume of literature has examined Irish experiences of migration to nineteenth-century Britain,^19^ and the burden the ‘Irish problem’ placed on welfare and penal institutions, only recently has attention turned to the high incidence of mental breakdown and confinement in asylums amongst Irish migrants to Britain after the Great Famine.^20^ This contrasts with the volume of research and literature on the experiences of Irish migrants in psychiatric hospitals in nineteenth-century North America and the Antipodes where rates of admission amongst Irish migrants were also high.^21^ Irish patients figure frequently as tensions arose between workhouses and asylums in negotiating the reception of pauper lunatics in Lancashire in the second half of the nineteenth century. The Irish were reckoned not only to be numerous, something underlined in admission figures, but also prone to particular manifestations of mental disorder, notably mania, insanity associated with drink, and general paralysis of the insane.^22^

These issues were highlighted in the annual reports of the Lunacy Commissioners, asylum reception orders, reports and patient case notes, newspaper accounts, Poor Law records and a unique set of notebooks produced from the late 1860s onwards by Lancashire County Council in its quest to establish the settlement of Irish patients for the purpose of chargeability, key sources upon which this article is based.^23^ A database compiled of samples of Irish and non-Irish patients recorded by place of birth in Rainhill Asylum's casebooks between 1856 and 1906, also provides rich evidence of variations between Irish patients and their non-Irish counterparts in terms of their familial situations, diagnoses and precipitating causes of mental breakdown.^24^ Our evidence demonstrates significant shifts in the relationship between Poor Law and asylum in response to the challenge of managing pauper lunacy across our period. During the 1850s and 1860s asylum superintendents and the Commissioners in Lunacy were reluntant to relinquish the treatment of mental illness to workhouse medical officers, as asylums were built in Lancashire and systems of care guided by the principles of moral management established. By the late nineteenth century, in situations marked by intense pressure on space and staff in workhouses and asylums, attitudes had shifted dramatically and were characterised by frustration on the part of asylum superintendents at what they perceived to be the common practice of Poor Law officials in dumping unwanted patients into asylums. These anxieties gained further force as incurable, chronic cases accumulated in all institutions.

By contributing in large numbers to the problem of long-stay patients in severely overcrowded asylums, Irish patients became emblematic of broader problems and pressures facing Lancashire's asylums and were depicted in newspapers and in official records as a vast burden in terms of the cost to the county. Historians have largely ignored the routes through which Irish ‘lunatics’ entered the asylum, or the ways in which Irish patients were passed, bartered and exchanged between the two sets of institution. This was part of the larger story of efforts to deal with the vast number of pauper admissions flooding asylums and workhouses nationally, which was particularly remarkable in London and the northern manufacturing districts during the second half of the nineteenth century. Such patients were not just seen as numerically overwhelming but also as the ‘worst material’ to use the term employed by Dr David Cassidy, Medical Superintendent of the Lancaster Asylum, in 1896; chronic cases, difficult to treat, who placed a great strain on institutions, and were less likely than other patients to be discharged back into the community and the care of their families.

## The Burden of Pauper Lunacy

Throughout the nineteenth century, the Lancashire Poor Law Unions contributed huge sums for the support of pauper lunatics—Irish and non-Irish—in asylums and workhouses. As early as 1844 Lancashire had the unhappy record of confining the largest number of lunatics in workhouses, some 369 compared with Middlesex's 266, although the numbers—even taking underreporting into account—were still relatively small.^25^ By December 1854 Poor Law Inspector, H. B. Farnell, reported that £174 11s 6d had been paid for the maintenance of patients in the Lancaster Asylum, and by 1866 it was estimated that Lancashire County paid £2,000 a year for the costs of asylum patients. Preston Poor Law Union claimed that expenditure had risen from £1,119 to maintain 58 patients in asylums in 1856 to £2,000 for 120 patients in 1866.^26^ The influx of pauper lunatics into asylums and chargeable to the county seemed to be limitless. In 1869 an article in the *Preston Guardian*, ever alert to the dangers of pauperism, Irish migration and the increase in local rates, claimed that ‘Pauper lunatics are increasing at the rate of about 200 a year in Lancashire’.^27^ Meanwhile, the *Pall Mall Gazette* estimated that there were 44,924 mentally ill patients supported by the rates in England and Wales. The article went on to allege that parish officers refused to send mentally ill inmates to the workhouses for ‘fear of patients attracting the attention of the Lunacy Commissioners’, who would insist on their removal to the asylum, the more expensive of the two options. Instead, it was claimed, the mentally ill were kept at home in the hope of concealing them from the Commissioners.^28^

**Fig. 1.**
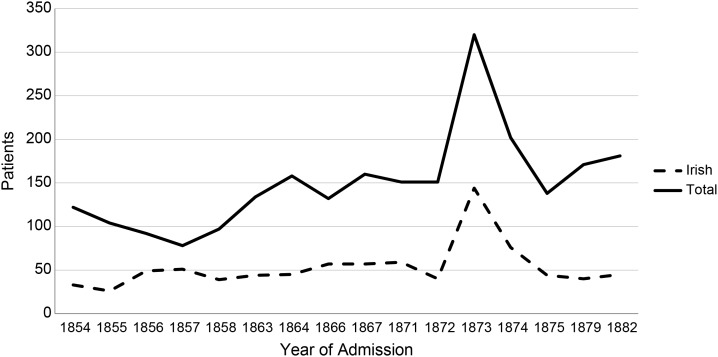
Number of Irish and non-Irish patients admitted to Rainhill Asylum, 1854–1882.

*Source*: Annual Reports, Rainhill Asylum, 1854–1882. The spike in the figures in 1873 results from the removal of patients from Rainhill to the newly opened Whittingham Asylum, which enabled Rainhill to admit a batch of new admissions in that year.

While the drain on Lancashire Poor Law Unions was substantial, the county's robust industrial and commercial development meant that it was better able than most regions to shoulder the expenses of pauper lunacy.^29^ It did not go unnoticed, however, that a substantial part of such costs was for the maintenance of Irish migrants in Lancashire's asylums and workhouses.^30^ The Irish made a dramatic impact on admissions to Lancashire's four public asylums: Lancaster (established 1816), Rainhill (1851), Prestwich (1851) and Whittingham (1873). Disembarking at Liverpool, many Irish migrants quickly entered the workhouse and then were transferred to the county's asylums. Rainhill, near Liverpool, bore the brunt of these admissions. By the late 1850s, Irish patients accounted for half of its intake; in later years this dipped to around a third of admissions, still a significant and troubling proportion of the total (see Figure 1). During the 1850s and 1860s, the Irish outnumbered English admissions in many years, and they consistently far outranked the admission of other nationalities to all four asylums. By the 1870s Irish patients made up around half of the resident population at Rainhill.^31^

Asylum doctors and the Lunacy Commissioners closely tracked the impact of migrants on patient figures, expressing alarm at the numerical onslaught represented by the Irish. By 1856, five years after Rainhill opened, its superintendent Dr John Cleaton reported overcrowding in ‘some rooms’; 25 per cent of the patients admitted in that year were Irish and he observed that 50 per cent of all admissions were in an ‘exhausted state of health’. Cleaton also commented that recovery rates were lower and death rates higher amongst patients ‘shattered in bodily health and condition’.^32^ In 1863, in the midst of the Lancashire Cotton Famine, it was reported that of the 138 patients chargeable to the county in the Lancaster Asylum, 90 were natives of Ireland. It was also noted how difficult it was to ascertain settlement and, after brief inquiry, maintenance orders tended to be imposed on the county. The report went on to point out that Ireland had excellent District Lunatic Asylums and urged legislation to ‘remove so heavy a burden from the county’.^33^ In 1868 Dr Thomas Lawes Rogers, Cleaton's successor at Rainhill, who held the post of superintendent between 1858 and 1888, noted that two-fifths of the asylum's inmates were Irish, compared with one-third in the previous year.^34^ Despite the fact that migration from Ireland had reduced to a steadier flow, as late as 1882 the Lunacy Commissioners were still commenting on the major contribution of Irish patients to the burgeoning populations of Lancashire's asylums.^35^ While the Liverpool authorities were entitled to repatriate the pauper Irish under Removal and Settlement legislation, the sheer scale of the ‘Irish’ problem during the Famine period and changes to legislation thereafter making it illegal to remove paupers with residency, meant that it was difficult to enforce the legislation.^36^

While most Poor Law assistance to the Irish was provided in the form of outdoor relief, they were also admitted in disproportionately high numbers to the county's workhouses. Estimates suggest that Irish and Scottish admissions—and the vast majority were Irish—accounted for an average of 20 per cent of Manchester's workhouse population during the 1850s.^37^ According to Liverpool's Select Vestry, there were still large numbers of Irish inmates in 1865.^38^ Many of the Irish admitted to workhouses ended up in their lunatic wards and of these a large proportion were transferred to the Lancashire asylums, a phenomenon that we will examine more closely in the following sections. Irish pauper lunatics were also placed into other asylum facilities, including Haydock Lodge private asylum, near Liverpool, which in 1846 had been licensed to take 400 paupers and 50 private patients. Though the number of pauper patients shrank in the 1850s when the new county asylums at Rainhill and Prestwich near Manchester first opened, it was utilised for many decades thereafter to absorb patients who could not be accommodated in the overflowing public asylums.^39^

The Irish also made up a hefty proportion of Lancashire's prison population, including many admitted under vagrancy legislation. In January 1851, Reverend John Clay, Chaplain at the Preston House of Correction, observed that the increase in committals for vagrancy—362 during the previous year—was due to the ‘influx of vagrant Irish’.^40^ The problem had been acute since 1847 and in July of that year, Liverpool Magistrate Edward Rushton, conceded that the Vagrancy Act was ‘a dead letter’ as Lancashire's prisons could no longer cope with the numbers, notably the Irish, committed under the legislation.^41^ During the nineteenth century, many Irish were transferred from local prisons and Broadmoor Criminal Lunatic Asylum to Lancashire's asylums, further boosting admissions of largely male but also female patients charged to the poor rate. For example, Elizabeth Wilson was moved from Kirkdale Prison to Rainhill on 18 May 1870. Prior to her offence of stealing a coat, she had lived in Manchester for thirteen years. Her parents had died in Ireland, and she had been widowed for three years. On admission to Rainhill, she was diagnosed as suffering from dementia and general paralysis.^42^ John Thompson, an ex-soldier born in Ireland, was sent to Broadmoor after his sentence for burglary in 1861, and thence to Rainhill in 1868, where he was kept at the expense of the poor rate. His initially violent and dangerous behaviour improved while at Rainhill and he was moved to Whittingham Asylum in 1873, presumably still at the county's expense.^43^

## Authority to Claim and Cure: The Workhouse and Asylum in the 1850s

After Lancashire's new public asylums opened in 1851 at Rainhill and Prestwich, one of the key concerns of the asylum superintendents and the Lunacy Commissioners was to urge the Poor Law guardians to relinquish their lunatics into the care of asylum doctors. As Felix Driver has noted, from the first ‘advocates of specialised asylum treatment had rejected other institutions, including workhouses and prisons, as inappropriate places for the care of the insane’.^44^ During the 1850s asylum superintendents, spurred by the spirit of reform in their new purpose-built asylums, argued that they and they alone were the fit and proper persons to treat lunacy, even in cases with a limited chance of improvement. They argued that all pauper lunatics currently housed in workhouses—both recent and chronic cases—should be transferred to the newly opened asylums. The opening of these institutions ushered in a phase of optimism and confidence about their ability to cure—or at least improve the conditions of their patients—and the medical superintendents of Rainhill and Prestwich Asylums rigorously opposed the efforts of the Poor Law authorities to cut costs by retaining cases of insanity in workhouses. However, over the course of the second half of the nineteenth century, ideas about where to house the vast numbers of insane paupers shifted dramatically. Numbers increased, so that by the late 1860s and 1870s asylums were overflowing with patients and struggling to admit new cases, and their superintendents flipped from urging the removal of lunacy cases from workhouses to actively discouraging this practice.^45^ They became preoccupied with the undesirable traits and hopelessness represented by patients sent from workhouses, and expressed frustration at what they saw to be the practice of the Poor Law in dumping large numbers of patients into asylums as their facilities were extended. These shifts were complicated by legislative changes, though these were not always responded to in practice, and by the slowly improving conditions and increased specialisation in treating lunacy in the county's workhouses. They also marked a sea change in the attitudes of the Commissioners in Lunacy from seeing themselves as ‘ambassadors of enlightenment and expertise’ after the passing of the 1845 Lunacy Acts to acknowledging the extreme pressures being placed on the asylum system by the 1860s.^46^

When Rainhill and Prestwich asylums first opened in 1851 large numbers of lunatic paupers were accommodated in local workhouses and the asylum superintendents, with the support of the Commissioners in Lunacy, attempted to relieve the workhouses ‘from a charge which ought never to have been imposed on them’.^47^ Over the next few years workhouse inmates exhibiting various mental disorders—recent, incurable and chronic—were ushered into the new asylums. Forty were quickly transferred from Manchester workhouse to Prestwich Asylum and by April 1852 Rainhill had accepted 46 admissions from Lancashire's workhouses. Within two years of Rainhill's opening, an article in the *Liverpool Mercury* noted that ‘The workhouses in the county of Lancashire, many of which, until the last year, contained numerous cases of insanity in its various forms, and more especially of dangerous epilepsy and idiocy, have now been in a great measure relieved’.^48^ The vast majority of the patients transferred, however, were chronic and incurable; only 12 of the 222 patients transferred into Rainhill Asylum from other asylums and workhouses in 1851 ‘afford[ed] much hope of recovery’.^49^ This was also the case at Prestwich where the Committee of Visitors soon became apprehensive about the institution becoming ‘so full of incurables that very few of the more urgent cases will gain admission’.^50^ At the same time, patients housed in licensed private asylums, including Haydock Lodge, were transferred to Rainhill and Prestwich. Of those admitted from workhouses into Rainhill Asylum, many were Irish migrants. The Rainhill Asylum Reception Orders for 1856 indicate that 90 per cent of Irish admissions in that year were pauper lunatics transferred from workhouses.^51^ The numbers remained high in the post-Famine period; between October 1865 and April 1866, 22 out of 31 Irish admissions to Rainhill Asylum (71 per cent) had been transferred from workhouses.^52^

Technically, under the 1842 Poor Law Order, paupers admitted to the workhouse who displayed signs of mental disorder were to be removed to the asylum within fourteen days though those who were not dangerous could legally be kept in the workhouse. The Order was often ignored, and the loophole referring to ‘dangerousness’ interpreted in a flexible manner. Asylum doctors, Visiting Committees and the Commissioners in Lunacy repeatedly cautioned the Poor Law guardians for retaining lunatics, including many who were deemed curable, in their workhouse wards. They stressed that early removal to an asylum was crucial in offering patients the best chance of cure or improvement, and in 1856 Rainhill's Committee of Visitors complained of delays in relinquishing pauper lunatics—in some instances the transfers took between 19 and 44 days.^53^ In 1857 Rainhill received 50 patients from the workhouse. In 44 cases, their average detention was 15 days—not bad given the limit of 14—but in individual cases this had varied from one to seven weeks.^54^ There were pragmatic, financial incentives involved too, a point not lost on Rogers at Rainhill, who argued in 1859 that the Poor Law authorities used the increasing pressure on asylums as a pretext for detaining lunatic paupers in workhouses where it was cheaper to maintain them.^55^

Already by the mid-1850s Rainhill and Prestwich Asylums were packed and new applications were being refused.^56^ One response to this was to petition to expand the existing asylums and, by the 1860s, to press for the establishment of a new asylum, an expensive option involving major capital investment. By 1858, proposals had been put forward to enlarge Prestwich and Rainhill Asylums.^57^ While considering the plans to enlarge Rainhill in January 1858, several county magistrates, reluctant to expend more on asylum construction, urged the asylum authorities to transfer incurable patients to workhouses in order to release space for the admission of new, more promising cases. They observed that ‘valuable space’ in Rainhill was ‘occupied by idiots and other inoffensive imbeciles, who are much fitter objects for a parish workhouse’.^58^ Given the level of overcrowding in Rainhill and in other asylums—in 1854 Medical Superintendent Dr Cleaton reported that the low number of admissions was due to the asylum being full rather than resulting from a decline in applications—this presented a practical solution to the problem of overcrowding and after 1857 the Lunacy Commissioners gave ‘qualified approval of the practice of sending certain cases to the workhouse’.^59^ Reluctantly, the asylum medical superintendents and Committees of Visitors at Rainhill, Prestwich and Lancaster agreed to transfer patients from their asylums to workhouses to facilitate the admission of new cases.

Asylum medical superintendents were, however, unwilling to pursue this practice on a larger scale and the negotiation of patient exchanges between the Poor Law and asylum authorities became acrimonious at times. When in 1858 the asylums' medical superintendents informed the county magistrates that only a few patients were suitable for transfer to the workhouses, they were accused of being too fastidious in their selection and of protecting their professional interests and status.^60^ Nor did the transfers significantly alleviate the pressure on asylums; in 1854 Dr Joseph Holland, Medical Superintendent at Prestwich Asylum found that the removal of several epileptic and demented patients to workhouses failed to release space for new admissions.^61^ As the Commissioners in Lunacy observed a decade later, for the policy to be successful, it would have to pursued on a massive scale,^62^ a point Dr Cleaton made in 1858 when he argued that to remove sufficient numbers of chronic patients from asylums, workhouses would be obliged to establish ‘chronic asylums’ to cope with the numbers.^63^

The Commissioners in Lunacy and asylum superintendents were very critical of Boards of Guardians who erected special and extensive wards in workhouses with the intention of retaining pauper lunatics, though, as Elaine Murphy has suggested, by the late 1850s, the Commissioners conceded that most workhouse lunatics were beyond cure.^64^ Negotiations on the transfer of chronic patients from Rainhill Asylum to Brownlow-hill workhouse illuminate the concerns and priorities of the different groups. In 1856 the Liverpool Select Vestry suggested that to avoid the expense of extending Rainhill, which was ‘often full’, chronic patients should be removed to Brownlow-hill workhouse.^65^ Previously, conditions in the lunatic wards had been criticised by the workhouse Medical Officer, Dr Robert Gee and the Assistant House-Surgeon, W. Eddowes and a series of improvements had been made in 1853 and 1855.^66^ By 1856, the Guardians felt they could reassure Rainhill's Visiting Committee that conditions at the workhouse had improved through ‘extensive alterations … the Vestry have now provided large, light and airy wards’ and they intended to appoint a medical officer to oversee these.^67^

While the Liverpool Guardians assured the Asylum Visiting Committee that they would transfer new and curable cases of mental illness to the asylum, they also indicated that they would retain ‘idiot’ patients in the workhouse wards and requested that harmless, hopeless and incurable cases in Rainhill Asylum be returned to the workhouse. Rainhill's Medical Superintendent, John Cleaton, had visited the workhouse wards and found them to be better than expected, and its Committee of Visitors had no objection ‘to allowing weakminded idiotic patients or bed-ridden incurable lunatics’ to remain in workhouse wards ‘when properly furnished, especially as it would afford more room in the County Asylum for those lunatics whose recovery might not be hopeless’. The scale of provision, however, set off alarm bells:

far exceeding anything that can probably be required for idiots or bed-ridden incurable lunatics, that it is in contemplation to retain there such lunatics as may be pronounced incurable by the Medical officers of the parish. … We would remark that lunatics are not, as a general rule, sent from the Workhouse to the County Asylum as soon as they ought to be;^68^ …

The workhouse was also noted as being unsuitable for patients who were suicidal, refusing food or melancholy, or patients deemed to be potentially curable or whose symptoms might be alleviated. Aside from incurable and harmless patients, the Visitors argued that the insane poor needed care in specially constructed asylums. Attendants in workhouses, they suggested, were ‘ordinarily paupers, unequal for duties’. Asylum patients had a better diet, were kept occupied and, most important, had an experienced medical staff to care for them.^69^ While much of the debate centred on the most suitable location for the management of chronic cases, at a point when asylum doctors were attempting to bolster their credentials and authority as experts in mental disorder, they were eager to retain control over the allocation of patients to the workhouse and asylum and to insist upon the primacy of the asylum in the management of pauper lunacy. Granting the Poor Law authorities and workhouse medical staff a more significant role in the process was regarded as potentially undermining this authority, and it put at risk one of the main tenets of treatment: early admission to asylums.

## An Unwilling Consensus: The Workhouse and Asylum in the 1860s

Asylum authorities were, however, fighting a losing battle and by the 1860s, as Murphy has shown, the Lunacy Commissioners were forced to concede that chronic asylum patients could be treated and maintained in workhouses.^70^ They continued, however, to object to the retention of new cases and of patients suffering from epilepsy and paralysis ‘who by reason of their violence and excitement not unfrequently [sic] most urgently require Asylum treatment’.^71^ Late removals could also trigger disagreements, and in 1868 Rainhill's Committee of Visitors criticised the Poor Law authorities for holding on to recent cases of mental illness until ‘they have become so violent and dangerous [as] to be quite unmanageable’.^72^ While asylum doctors continued to repeat the mantra that all patients should be housed in asylums, the logistics of managing so many patients made such calls unworkable.

The 1862 Lunacy Acts Amendment Act facilitated the transfer of chronic, harmless asylum patients to workhouses subject to the agreement of the relevant Poor Law Guardians and the Poor Law Board.^73^ It also specified that asylum doctors would be responsible for the selection of patients, therefore legally establishing them as ‘gate-keepers’ of patient exchanges. Following the passage of the Act, asylum superintendents were assured that Poor Law Guardians would be required to construct separate wards in their workhouses with appropriate furnishings and patients provided with a diet and outdoor exercise and recreation analogous to that of an asylum. Qualified medical attendants were be appointed to oversee the workhouse lunatic wards, and medical registers and other administrative records, kept by all institutions licensed to care for the mentally ill, were to be properly maintained. The Commissioners in Lunacy were also granted powers to approve workhouses for the reception of asylum patients.

Although disappointed by the Act, as it undermined the pre-eminence of the asylum in the management of mental illness, the Commissioners in Lunacy were insistent that these regulations be adhered to. The Committee of Visitors at Lancaster Asylum concluded that few workhouses would meet the standards, and their reservations were well founded; the passage of legislation did not resolve the difficulties surrounding the transfer of asylum patients to workhouses.^74^ For example, in 1866, the Visiting Committee at Prestwich Asylum entered into negotiations with the Guardians of Bolton Poor Law Union for the reception of asylum patients into the workhouse under the 1862 Act. Initially satisfied with conditions in Bolton's workhouse lunatic wards, the Commissioners in Lunacy supported the proposal. A year later, however, they had withdrawn agreement; the Crown legal officers had advised that the legal position of the asylum Visiting Committee, the Guardians and others in relation to workhouse patients needed further legislative definition. The proposal was abandoned and instead plans to expand Prestwich Asylum were pursued.^75^

Other problems arose: the Commissioners in Lunacy were powerless to compel the Poor Law Guardians to improve conditions for pauper lunatics in workhouses, and, when inspecting workhouse lunatic wards, they found great variation in standards. In 1869, they reported fairly positively on the West Derby Union workhouse in Liverpool where 132 pauper lunatics were housed; the furnishings were comfortable, the diet liberal and the attendants competent. Yet, they also found recent cases of lunacy waiting to be transferred to the overcrowded Lancashire asylums.^76^ The Commissioners remained uneasy about the practice of relieving pressure on asylums by transferring chronic patients to workhouses instead of building new asylums or expanding existing ones.^77^ This unease was heightened after the passage of the 1867 Metropolitan Poor Act, which allowed for the transfer of lunatics, along with children and fever and smallpox cases, to new, separate, institutions under central control. The act further diminished the Commissioners in Lunacy's authority, and, though the 1867 Act was initially confined to the accommodation of pauper lunatics in the Metropolitan Asylum District, for the Lunacy Commissioners it further undermined their position and ‘marked the triumph of the Poor Law Board over the Lunacy Commissioners’ in the management of pauper lunacy.^78^

Disputes about the removal of Lancashire asylum patients to workhouses rumbled on but they lost some of their urgency and conviction as the pressure on asylums continued to increase. The addition of further asylum accommodation also failed to relieve the situation. Rainhill, originally built to accommodate around 400 patients, opened an annexe in 1886 to take a further 1,000 patients. This too was soon filled to overflowing. In December 1869, Rainhill, Lancaster and Prestwich asylums, which between them contained 2,670 patients, were all reported to be full.^79^ Building and then extending asylums was an expensive business and in 1869 an article in the *Preston Guardian* reported that Lancaster Asylum had cost the ratepayers some £148,000, Rainhill £113,000 and Prestwich £120,000, while the proposed new asylum at Whittingham would cost £120,000. The burden of provision, the article went on, was exacerbated by those ‘imported mad into Lancashire’.^80^ The county workhouses were also under pressure; a further 4,800 lunatics—‘a great many … who should not be there’—were housed in them.^81^

Despite the pressure on the workhouses and their reservations about the appropriateness of workhouse facilities, the increasingly desperate Lunacy Commissioners advised asylum superintendents to make greater use of them. In April 1868 the Lunacy Commissioners recommended that the Visiting Committee and the Medical Superintendent at Lancaster Asylum consider removing ‘old chronic cases’ to the workhouse. In July 1868, the *Liverpool Mercury* noted that Brownlow-hill workhouse was overcrowded,^82^ and in the following year, they reported on an exchange of chronic patients from Rainhill Asylum for urgent cases in West Derby Union workhouse.^83^ The sheer scale and pressure on the Lancashire asylum system was underscored in 1869 when the Whittingham Committee, established to oversee the construction of a new asylum and comprised of local magistrates, anticipated, even as it was being planned, that the new asylum would not be large enough to cope with predicted admissions. The Committee suggested that further arrangements be made with local workhouses. In December, the Committee recommended that 450 patients ‘of the chronic and orderly class’ in Rainhill, Lancaster and Prestwich asylums be transferred to the lunatic wards of the county workhouses; the members specifically proposed that 100 patients be sent to Bolton workhouse. The Commissioners in Lunacy objected to the recommendation on technical grounds related to securing payment, and when this problem was resolved, the Bolton Poor Law Guardians found that dietary requirements specified by the Commissioners for workhouse patients were too expensive and once again the proposal fell through.^84^

During their deliberations, the Whittingham Committee held up Salford Hundred workhouse as an example of a union that had adopted ‘an enlightened policy’ by improving workhouse facilities for pauper lunatics and commended the Guardians at Bolton, Oldham, Manchester and Chorlton for introducing a similar policy. They claimed that as a result Prestwich Asylum ‘was mainly filled with patients of the violent, or, at any rate, of the early stage of insanity’ and not with chronic cases all of whom had been transferred to workhouses.^85^ In Liverpool, however, Brownlow-hill workhouse had not been enlarged; the building did not have sufficient space in its lunatic wards to accept patients and, as a result, 100 patients in Rainhill Asylum of the ‘quiet and orderly class’ suitable for transfer remained in the asylum.^86^ Liverpool, more than any other part of the county, was perceived to be under the greatest pressure, and its Select Vestry was paying the largest portion of its lunacy expenses for the maintenance of patients in private asylums such as Haydock Lodge which charged fees of around 15s per patient per week compared to 7s to 9s in the public asylums. Liverpool was also obliged to expend large sums on maintaining vagrant lunatics—estimated to cost £12,000 to £14,000 a year—and as the county council notebooks show, many of these were Irish.^87^ The large numbers of ‘non-settled pauper lunatics’—480 in the county—was linked directly to Lancashire's proximity to Ireland and it was claimed ‘steamers from Dublin’ brought over lunatics who were then ‘left in the streets of Liverpool’.^88^

The settlement of many such patients could not be traced. In December 1866 at the Annual General Session for the county of Lancashire magistrates noted that between 430 and 450 patients in the county asylums were paid for by the county—330 were Irish—and the ‘county were paying for patients it ought not to be’. ‘It was a curious fact that out of the 430 or 450 patients there were only 30 English, the rest belonging to other countries. Besides Irishmen, they had Dutchmen, Chinamen, Scotch, and natives of almost all countries in the world.—(Laughter.)’ Although such comments provoked amusement, the meeting revealed that costs for lunacy were amongst the largest charges imposed on the county rates; the total figure for new asylum building and the maintenance of existing asylums was estimated at £9,084 for the period November 1866 to August 1867.^89^ As a result of these concerns, in January 1867 the General Finance Committee of the Lancashire County Council passed a resolution to place all lunatics in Lancashire asylums, who were to be maintained at the cost of the county, into two classes; class 1 patients had no settlement in England and Wales and included Irish-born patients, who were to be maintained at the cost of the county, and class 2 included patients with settlement. The names and details of these patients were recorded in notebooks, which the Finance Committee maintained into the 1890s although with less rigour than in the early years.^90^ This initiative—while apparently largely a recording device rather than a spur to changes in practice—was indicative of the level of anxiety provoked by the cost of maintaining pauper lunatics, notably recent arrivals. The County Council notebooks also highlight the rapidity with which Irish migrants were moved from the workhouses of Lancashire into the asylum system.

Once such case, Francis Burns, was admitted on 17 June 1873 to Rainhill Asylum, only a few days after his arrival in Liverpool. A 42-year-old single man and labourer, Roman Catholic Irish, Burns had been taken in from the vagrant sheds to Liverpool Workhouse as a lunatic on 6 June. He had no known relatives in England. On admission to the Asylum he said he could perform miracles and ‘cure all diseases by putting his hands upon the persons’. He was described as being very rambling and excited, and had difficulty sleeping. His bodily condition was said to have improved while he was in the asylum although mentally he remained the same. He continually insisted that he was ‘going home’. He suffered much from ‘coughing’ and ‘exhaustion’, and died in Rainhill a few years later, in December 1879.^91^ Another Irish patient, Thomas James, was sent to Rainhill by the Township of Toxteth Park on 4 September 1873. He had first arrived in England in May 1871 and moved in and out of Toxteth Park workhouse, being admitted in October 1871 and then again in May 1872. He was still a patient in Rainhill Asylum in February 1880.^92^

As shown above, the presence and cost of Irish patients captured the interest of the local press, as did the additional demands placed on institutions for special provision for Roman Catholic chaplains and services, a further charge and inconvenience triggered largely by the Irish presence. In 1872, a year before Whittingham Asylum was due to open, an article in the *Preston Chronicle* questioned whether it should be designated a Roman Catholic institution, given the enormous number of Catholic patients in the county. A letter written by J. B. Booth, chairman of the Lancashire magistrates, reported that no chaplains were currently being paid for their services in tending for 821 Catholic patients in the Lancashire asylums and suggested that Whittingham, set to cater for 1,000 patients, could operate most efficiently as a dedicated Roman Catholic asylum which would also allow for a chaplain to be properly remunerated.^93^ In 1875, the Lunacy Commissioners observed that Irish patients boosted the proportion of Roman Catholics in Lancaster Asylum, and their numbers created practical difficulties for the provision of Mass, although a priest had been brought in to hold services there. In 1888, the Lunacy Commissioners criticised Lancaster for its failure to provide amenities for Roman Catholic services commenting that it was the ‘only one of the Lancashire asylums without such regular service’. At Prestwich Asylum, they observed that the large number of Roman Catholic patients attending mass in the ‘old hall’ in 1889 ‘gave an idea of how many Irish are there’.^94^

## ‘Fitter Objects for a Parish Workhouse’?

Irish patients, like Thomas James, made a significant contribution to the accumulation of chronic long-term patients. As Dr Rogers noted for Rainhill Asylum in 1870, the Irish ‘have steadily increased year by year in the residuum of incurables’.^95^ The type of Irish patients admitted—physically decrepit, extremely poor, isolated from family and friends and suffering from chronic illness—meant that they required more nursing and care. Many remained in institutions for years, even decades, until their deaths from disease or old age, or transfer to another institution.^96^ Across our sample years—1856 to 1906—Irish migrants were more likely to be single and had fewer family members and other resources to draw on; married patients were statistically under-represented among Irish admissions.^97^ Half of Irish male admissions to Rainhill were single compared with 40 per cent of non-Irish admissions. For women, there was less variation—about 40 per cent of all female admissions were single, and many of these—Irish and non-Irish—were domestic servants. However, a far higher number of Irish patients were widows, who constituted a highly vulnerable group; 17.4 per cent Irish compared with 11.6 per cent non-Irish. Like Elizabeth Wilson, discussed above, these women seem to have lacked family support, were otherwise isolated following the deaths of their husbands and were admitted rapidly to workhouses and asylums. Sarah Murphy, aged 80, was found wandering the streets by a policeman, who brought her to Liverpool workhouse, and from there she was transferred to Rainhill Asylum on 19 July 1870. A widow, who appeared to have no friends, was described when admitted as being on ‘her last legs, she has a troublesome cough. … There are various bruises about her face & body’. She was also noted to be disruptive, violent in her language, stripping off her clothes and trying to escape, and fancied ‘that she sees her grandchild when she looks at herself in the mirror’. Sarah gradually faded away, was dosed daily with brandy, ‘which she appreciates knowingly’. She remained ‘restless & scolding’ until her death from bronchitis and senile decay in June 1872.^98^

John Walton's findings with respect to the textile areas of Lancashire and Elizabeth Malcolm and Angela McCarthy's conclusions about Irish admissions to New Zealand asylums and during the gold rush era in Australia suggest that families tended to look after their own as far as possible before consigning them to an asylum, or at least maintained some form of contact with them. In contrast, Irish patients at Rainhill tended to be isolated from family and kin.^99^ Admission certificates noted time after time that Irish patients had ‘no friends’, ‘nearest relative unknown’ or that the nearest relative was in Ireland. Other Irish patients were ‘found wandering’ or ‘previous abode not known’; in 1856, 45 per cent of patients were described in these terms. This isolation did not diminish over the decades; in 1873, the proportion so identified was 46.8 per cent.^100^ The social isolation of the Liverpool Irish admitted to asylums contrasts starkly with Belchem's account of the building of an Irish community and support system in Liverpool, through the church and other agencies. The findings here accord more with Anderson's observations for Preston, which showed the isolation of many Irish in times of sickness or unemployment.^101^

Irish patients also were less likely to be discharged improved or recovered. This was particularly striking with respect to patients resident in the asylum for over ten years and amongst female admissions. They were more liable than non-Irish patients to suffer from chronic and incurable disorders, including dementia and general paralysis. For example, across our sample years, 14.3 per cent of married and 13 per cent of single, Irish female patients were diagnosed with dementia compared with 9 per cent and 11.7 per cent of non-Irish female patients. The rates of diagnosis were also higher among Irish men; 22.5 per cent of single and 20.2 per cent of married Irish patients were labelled as suffering from dementia compared to 20.8 per cent of single and 15.2 per cent of married non-Irish patients. The label was used flexibly and patients diagnosed with dementia included those suffering from various stages of general paralysis and epilepsy, progressive disorders that resulted in the obliteration of patients' intellect and a steady decline after years of illness. The perception of the Irish as being of weak intellect was fuelled by reports of alarming rates of pauper lunacy in Ireland compared with England.^102^ Irish patients were also perceived to be especially vulnerable to general paralysis of the insane, an ever more frequent diagnosis in the late nineteenth century. While reported to be free from the disease in rural Ireland, once ensconced in Liverpool, facing the temptations of city life, the Irish, according to the doctors treating them, became vulnerable to a disease that was incurable, on the increase, and which contributed to low discharge and high mortality rates.^103^

Large numbers of the Irish patients admitted to Rainhill Asylum fell into the cohort often regarded as better suited to workhouse confinement. Yet, Irish patients in Rainhill Asylum were not moved to Lancashire workhouses in great numbers and instead they remained entrenched within the asylum system. Many eventually died in Rainhill. Single and widowed Irish women were especially subject to this fate: 47.2 per cent of single and 66 per cent of widowed women across our sample years died in the asylum. Irish patients, without settlement, evidence of residency in the parish and family support networks, were particularly liable to be moved elsewhere and in the case of Irish patients this was often to Whittingham asylum after it was opened in 1873 for the reception of 1,100 patients (an annex was added in 1883 for a further 674 patients).^104^ The likelihood of transfer was related to the absence of family support networks; the Commissioners in Lunacy advised the Lancashire Asylum Visiting Committees that when selecting patients to be transferred to Whittingham and Lancaster Asylums they should send patients who were seldom or never visited by relatives while recent cases should be kept ‘nearest their homes’ and relatives at Prestwich and Rainhill.^105^ For some patients, it was commented in 1884, ‘it is of no moment in which asylum they be placed’.^106^

Among chronic patients, it was more common for non-Irish patients to be transferred to workhouses while Irish patients often ended up in Whittingham asylum. For example, five (14.7 per cent) of the 34 non-Irish patients admitted to Rainhill in 1866 were eventually transferred to workhouses; two were suffering from dementia and a third patient—Christina McDonald—was said to be a ‘simple, half-imbecile sort of girl’.^107^ McDonald, a 19-year-old unmarried English servant, was admitted to Rainhill with mania in May 1866. She was delusional and imagined that she saw ‘birds dressed in fantastic clothing and that she hears bands playing’. She experienced several ‘hysterical’ attacks and having spent almost exactly five years in the asylum, she was transferred to Riverford workhouse in North Yorkshire as incurable and harmless.^108^ In the same year, 24 Irish women were admitted, but none of them were removed to workhouses though two were subsequently transferred to Whittingham Asylum. One patient thus removed was Catherine Riley and in several respects her case was similar to McDonald's. Riley was also single, aged 20, though—in contrast to McDonald—she was described as a ‘prostitute’. Riley was admitted to Rainhill in April 1866, reported to be suffering from mania and delusional. Like McDonald, she sometimes became agitated and her mental condition did not improve; she could not ‘tell where she was born’. Unlike McDonald, however, having spent six years in Rainhill it was decided in May 1873 to transfer Riley to Whittingham Asylum.^109^

The reluctance to transfer Irish patients to workhouses may have been bound up with their reputation as difficult and unmanageable patients as well as their isolation from family and friends. While Irish patients often suffered from chronic disorders, they were noted in admission certificates and case records to be excessively disturbed, unruly and volatile, an association encouraged by traditional stereotypes of the Irish as excitable, bellicose and wilful and further fuelled by reports of unruly behaviour, fighting and high crime rates in the local press. This reputation was reflected in the diagnoses assigned to Irish patients. Mania was the most common form of mental disorder among all asylum patients, and in Rainhill Asylum it was diagnosed in 20 per cent of non-Irish patients. In contrast, over half of all male and female Irish patients were diagnosed with mania, an extraordinary difference.^110^ Mania was associated with incredible energy and strength manifested in violent, oftentimes unmanageable, outbursts, even among patients who were described as being weak and in poor bodily health. Time and again violence, dangerousness to others and frightening physicality was reported in the admissions certificates and case books amongst Irish patients: ‘wild and furious’, ‘strikes anyone in his way’, ‘raging violently’, ‘threatens each person in charge of him’.^111^ This perception of the Irish as more disruptive, violent and in need of additional management and resources among asylum medical superintendents may have mitigated against the identification of them as ‘harmless’, ‘quiet’ and consequently suitable for workhouse accommodation.

## The Close of the Nineteenth Century

By the late nineteenth century, the pressure of admissions on the Lancashire asylums was immense; the annexes at Rainhill and Prestwich and the new asylum at Whittingham rapidly filled with patients, often chronic, long-stay pauper lunatics many of whom were transferred from workhouses. In this context, the perspective of the Commissioners in Lunacy and asylum superintendents shifted again. By the 1860s they had been forced to move away from their initial insistence—something expressed with particular strength in the 1850s—that all pauper lunatics should be housed in asylums and developed a more pragmatic and flexible position. By the final decades of the century they were expressing resentment about the numbers of patients being sent on from workhouses, complaining that these were largely chronic, hopeless cases whose accumulation prevented the admission of curable lunatics, inflated the asylums' mortality figures and suppressed recovery rates. The workhouses, however, appear to have been successful in removing their patients and by 1890, the Medical Superintendent at Prestwich Asylum reported that a comparatively small number of Lancashire's 9,500 pauper lunatics were housed in workhouses or with relatives. In 1861–71 the various Union workhouses took charge of 40 per cent of lunacy in the county, in 1871–81 36 per cent and between 1881–91 this fell to 22.5 per cent.^112^

With pressure on asylums mounting relentlessly, frustration was expressed about the types of patient being transferred from workhouses. Dr H. Rooke Ley, Superintendent at Prestwich Asylum, warned in 1871 that as long as the system of exchanging chronic asylum patients for ‘dangerous and destructive’ workhouse patients continued, the asylum death rate would remain high, the recovery rate low and the ‘tranquility of our wards … considerably interfered with’.^113^ The large numbers transferred from workhouses to asylums exacerbated the deteriorating situation in asylums; in 1883, 60 patients from Oldham workhouse and a further 80 from Bolton workhouse were removed to Lancaster Asylum and, in this instance, the Commissioners were particularly aggravated as the medical officer at Bolton had previously certified these patients ‘as proper patients to be kept in a workhouse’.^114^ In 1885 Liverpool Select Vestry were accommodating between 650 and 670 lunatics in asylums.^115^

The contribution of Irish workhouse patients and vagrants to this problem was significant. This picture is borne out in the Lancashire county council notebooks; for example, on 14 August 1873 Malachi Hart, a 53-year-old Irish married labourer, was admitted to Rainhill suffering from dementia and general paralysis. According to the notebook he had been living in the vagrant sheds in Liverpool since 5 August but little else was known about him. While in the asylum, he was ‘shaky on his legs’ and exhibited ‘evident symptoms of GP’. He ‘sank rapidly’ and died in November 1873.^116^ In 1896 at least 22 out of the 53 Irish admissions to Rainhill (41.5 per cent) were described as inmates of the workhouse, two more patients were vagrants and a further two had been transferred from prisons.^117^ Irish patients continued to be isolated even as close-knit Irish communities were being created in Liverpool; in 1896 35.6 per cent of Irish patients were described in Rainhill Asylum admission orders as having few family support networks.^118^

Asylum Visiting Committees and medical superintendents were particularly resentful when large numbers of workhouse lunatics, many Irish and suffering from ‘chronic insanity in its most hopeless forms’, were deposited in their institutions when new facilities were made available.^119^ When a new annexe opened at Prestwich in 1884 it rapidly filled to reach its capacity of 840 patients. Some 70 per cent of these, according to the Commissioners in Lunacy, had been transferred in batches from workhouses:

and the patients so transferred are for the most part imbecile, who had long resided in the lunatic wards of their workhouses and had been considered and so reported over and over again to our office, as suitable cases for Workhouse treatment. We cannot refrain from expressing a strong opinion of the impolicy of filling up asylums, constructed at great cost and working with an expensive staff, with a class of lunatic who do not need Asylum treatment, and who may be maintained more cheaply and with more happiness to themselves in the workhouses of their own districts.^120^

Dr Rooke Ley at Prestwich observed in 1889 that the larger workhouses in the Lancashire district accommodated 1,300 lunatics, which he characterised as ‘a reserve of mental disorder from which, in a large measure, this Asylum draws its supplies’. He went on to note that: ‘From time to time these workhouses empty their surplus population into our Wards and it is mainly from them that we have received during the past year, and in each year since the opening of the Annexe, so many cases of chronic insanity in its most hopeless forms.’^121^ The transfer of chronic workhouse patients into asylums continued to depress recovery rates and increase the death rate, particularly as large numbers were old, infirm and suffered from general paralysis and other incurable conditions.^122^ In 1884 Mary Lennon, diagnosed with ‘senile dementia’, was moved from Walton workhouse to Rainhill Asylum. At the time she was aged 84, a widow in feeble health but still noisy and quarrelsome. She was described in the casebook as ‘a very old woman—very deaf—and unable to give any account of herself except that she was born in Ireland’. Though ‘a grey haired withered old woman, in feeble health’, the case notes asserted that she constantly attacked the other patients. She died in Rainhill in January 1886.^123^ In the case of Caroline Whittle, a 25-year-old Irish woman admitted to Rainhill in February 1896, a history of hereditary insanity was identified; both her grandmother and her paternal uncle had been patients in Rainhill. Diagnosed with mania, she was described as violent, disruptive and suffering from delusions. Throughout her nine and a half years in the asylum, she was variously described as ‘noisy’, ‘abusive’, ‘restless’ and at times ‘violent’. Her physical condition was also very poor and she eventually died of tuberculosis in August 1906.^124^

In Lancashire, as patients were moved into asylums, the proportion of patients accommodated in workhouses declined significantly—from 37.5 per cent in 1880 to 24.4 per cent in 1890—and some asylums such as Lancaster, Prestwich and Whittingham refused to accept more chronic cases either from workhouses or other asylums.^125^ The Lunacy Commissioners and asylum superintendents claimed that the introduction of the four-shilling grant-in-aid in 1874 provided Poor Law authorities with a financial incentive to transfer patients to asylums and further contributed to the problem of overcrowding and the accumulation of incurable patients in asylums. Robert Ellis has, however, shown that the contribution the grant made to swelling the size of the chronic asylum population was less clear-cut and most likely contributed little to the well-established patterns of phenomenal growth and overcrowding. As Ellis argues: ‘By the time of the grant's introduction in 1874, the complaint that the asylum was becoming a “dumping ground” had become a hackneyed feature of the Commissioners' and Superintendents' annual reports’.^126^

Still attempts were made to transfer chronic patients from asylums to workhouses, though some of these patients were subsequently returned to newly opened asylum annexes and other asylums. Reporting on the Whittingham Asylum in 1886, the Commissioners in Lunacy observed that of the 30 asylum patients transferred to workhouses, ‘not one had been sent back here’ suggesting that this was not always the case.^127^ In 1893, once again faced with a crisis of accommodation in the Lancashire asylums and awaiting a decision on a proposal to construct another asylum, the Commissioners reported on a project to accommodate 80 or 90 patients at Rochdale workhouse under the 1890 Lunacy Act. By April Prestwich Asylum had transferred the first ‘batch’ of 20 patients and removals continued until 80 patients—40 male and 40 female—were transferred.^128^ This arrangement was intended to bridge the gap in accommodation until the fifth Lancashire asylum at Winwick opened in 1903.^129^

In the final decades of the century, asylum doctors found it increasingly difficult to negotiate the transfer of asylum patients to workhouses, although some Poor Law Unions had significantly expanded their facilities. In the 1870s, Brownlow-hill workhouse developed fully-fledged lunatic wards with attendants and superintendents, while the Select Vestry had established a branch at Dingle Mount for the treatment of female epileptics and lunatics.^130^ At Prestwich Asylum, the medical superintendent noted in 1887 that for the previous eighteen months they had been very successful in clearing chronic patients to workhouses and elsewhere. However, the workhouse accommodation was now full and the Poor Law Guardians refused to accept more patients. The Visiting Committee at Whittingham Asylum, which had been used to house ‘chronic’ incurable patients, many of them Irish and transferred from Rainhill and Prestwich, were more successful, having been encouraged by the Commissioners in Lunacy to remove chronic patients to workhouses after 1884; in April 1885 the transfer of nine patients to Brownlow-hill Workhouse was successfully negotiated.^131^ In 1899, an acute ward was added at Whittingham Asylum for recent cases of insanity to ‘favourably influence the recovery rate’.^132^ The opportunities for patient exchanges were substantially diminished by the early twentieth century. In 1906 Liverpool Select Vestry reported that ‘three-fourths of the certified lunatics were maintained in the asylums and the remainder in the workhouses’, and there was a proposal to extend workhouse accommodation.^133^ In 1907 the lunatic wards at Mill Road workhouse were overcrowded but when asylum accommodation was sought, the asylums at Winwick, Lancaster, Rainhill, Prestwich and Whittingham reported that they were full.^134^ A new asylum was under construction at Whalley, while the West Derby Unions were forced to place pauper lunatics in asylums outside the county.^135^

## Conclusion

Writing in the mid-1880s, the Commissioners in Lunacy reflected on the difficulties workhouse patients presented the asylum system; on the one hand, they argued, it was ‘undesirable’ to have asylum beds occupied by chronic, incurable cases while new cases could not be admitted; on the other hand, they insisted that dangerous and recent cases should not be retained in workhouses in the expectation that they would quickly recover.^136^ Their conclusions represented a fundamental shift away from an early, almost ideological stance, adopted in the 1850s that stressed the importance of asylum treatment, specifically moral management, for all forms of lunacy. Yet, in spite of embarking on a massive asylum-building programme, nationally and in Lancashire, the asylum system was unable to withstand the pressures of admissions—Irish and non-Irish—in the late nineteenth century and medical superintendents and the Commissioners were forced to relinquish some control over pauper lunacy to the Poor Law and workhouses. While in the 1860s vestiges of resistance were still evident among some medical superintendents and the Commissioners, legislative change and the sheer scale of the problem of overcrowding faced by asylums, especially in Lancashire, forced them to adopt a more pragmatic response. This gave way to outright frustration and anger in the final decades of the century as they found it increasingly difficult to remove chronic patients from asylums to workhouses thereby allowing for the admission of new cases, while additional chronic patients—some of whom had been in asylums previously while others were determined to be beyond asylum treatment—were dumped into expensive asylum accommodation. This was another example of the medical superintendent's inability to ‘influence significantly the process of confinement’.^137^ By the final quarter of the century, Lancashire had one of the largest systems of confinement in the world, and the second largest in Britain after Middlesex, but still it could not withstand the pressures of pauper admissions. In 1901 the Commissioners in Lunacy reported that while the asylum population of the county had quadrupled over the last 40 years, the numbers of insane inmates in workhouses had ‘increased but little’.^138^

In contrast to the findings of David Wright and other studies of the history of confinement in England and Wales, which have emphasised the importance of the family in the admission of patients to the asylum, our study, drawing on record linkage between asylum records and the county council notebooks, stresses the frequency of admissions via local workhouses. As an editorial in the *Liverpool Mercury* observed in 1869 ‘About one-half of the accommodation at Rainhill was occupied by persons—a large proportion of them from Ireland—who were brought over to Liverpool, and there turned adrift, until they were taken to the workhouse, and from thence to the lunatic asylum’.^139^ Yet, in spite of working in extreme and stressful conditions, when transferring patients to workhouses, medical superintendents were remarkably careful in their selection and much depended on the reputation patients had for tranquillity and manageability. The Irish, perceived to be especially troublesome and challenging to asylum regimes of order—an important consideration when managing these huge institutions—and a significant financial drain on Poor Law and asylum resources, were not identified as a group particularly suitable for admission or readmission to workhouses. Instead, the social isolation that often precipitated their initial entry into an institution also contributed to their transfer to asylums such as Whittingham and later Winwick, which catered not only for chronic patients but also for patients with limited support networks, alone and disconnected.

## Funding

This work was supported by the Wellcome Trust awards [089220/A/09/Z to H.M., 089220/B/09/Z to C.C.].
